# Surgery in San Antonio

**Published:** 1886-06

**Authors:** R. Menger


### SOME SURGERY IN SAN ANTONIO.

Editor Daniel's Medical Journal.

During the last few weeks a great deal of operative surgery has been done in San Antonio. This report will only concern the most interesting cases, and such as came under my immediate observation.

Dr. P. Ornelas a few days ago, performed *laparotomy* in a boy, aged 14 years, for abdominal tumor. The tumor seemed to be of traumatic origin. I will state, as nearly as I can, the particulars. The boy had been injured by a horse in the right hypogastric region over a year ago. Soon after the injury a swelling was noticed, but it seemed as if the parents had paid but little attention to it, until of late, when a well defined tumor could be felt through the abdominal walls. The tumor was slightly moveable and could be traced below in the hypogastric space and extending to the linea alba and iliac fossa. There was slight fluctuation on palpation, and in the upper region near the diaphragm, the tumor seemed to be nodulated and of hard consistency, giving the appearance as if it had connection with or originated from the omentum. The diagnosis was divided between sarcoma and floating kidney. On opening the abdomen, some serous fluid escaped and the tumor was examined with the hand. It was a solid, partially nodulated, sarcomatous tumor, nearly the size of a small childs-head, extending beyond the intestinal track on the right side down to the spinal column. It was adherent, at different places, to the intestines, and to the peritoneum parietale. Entire excision, or enucleation could not be done on account of the many adhesions and the extremely emaciated condition of the child. Dr. Truehart of Galveston, as guest, and Drs. Herff, Graves, Favre and myself were present and assisted. [It is to be inferred that the tumor was, then, partly removed; the writer failed to give the result of the operation. Ed.]

Another interesting case concerns a patient of Dr. Adolf Herff, afflicted with vesico vaginal fistula. The orifice was large enough to admit three fingers. It was situated above the collum vesical, leaving at this place only the urethra and a small part of the bladder intact. The first attempt to close this large defect, was not satisfactory, due mostly to improper light. After about four weeks, Dr. A. Herff made a similar operation, and this time in the hospital, where more artificial light was had. The result was very satisfactory; the patient being able to urinate freely. Each operation lasted two hours; it was neatly done.

RAIL ROAD INJURY.—A Mexican, aged about 38 years, was run over by a work-train; a number of car-wheels passed over his legs. He was sitting upon the side of a flat car, with his legs hanging over the edge, and in passing,—a plank or post [?] near a cattle-guard, his right leg struck against the vehicle and knocked him from the car directly under the wheels. He was removed to San Antonio for treatment, and arrived about three hours after the injury occured. It was nearly midnight, when I took charge of the case in the hospital. Both legs were torn to pieces just above the ankle, the achilles tendon only and his boots keeping them in connection with the feet. The bones of the right leg were fractured to splinters upwards to the gastrocnemius; and the soft parts, and tibia of the left leg were also crushed to jelly to the ankle joint.

As it was an urgent case, and having no other instruments at my command, (it being night and, my amputating cases were locked up in a drug store), with a common pocket-case and a good steel saw, and with no other assistance than Dr. J. Favre, (who luckily was at my house the same night), I concluded to amputate at once. The right leg I amputated in the upper, and the left one in the lower third. The Sisters of Charity assisted in holding the lights and forceps in ligating the arteries, and Dr. Favre gave chloroform. The unfortunate man survived the operation, but we had to inject five times at intervals a hypodermic syringe full of whiskey to keep his power up. The man was very anæmic, but there was, as usual in such injuries, but little hemorrhage.

The next morning, to our surprise, we found the patient quite cheerful, with pulse feeble and accelerated, and complaining but little. On the third day, he complained somewhat of his throat and mouth and we thought trismus would be the result. I had given him a morphine injection before and after the third day. After the fifth day he could swallow better and felt in general all right. On the twelfth day, the bone of the left leg was protruding a little caused by sloughing of a small part of cuticle, and re-amputation was feared. Two weeks later however, the fiaps granulated satisfactorily and the stumps of both feet will now in the fourth week, soon be completely cicatrized.

